# Discrepancy Between In-clinic and Haemagglutination-Inhibition Tests in Detecting Maternally-Derived Antibodies Against Canine Parvovirus in Puppies

**DOI:** 10.3389/fvets.2021.630809

**Published:** 2021-03-01

**Authors:** Paola Dall'Ara, Stefania Lauzi, Joel Filipe, Roberta Caseri, Michela Beccaglia, Costantina Desario, Alessandra Cavalli, Giulio Guido Aiudi, Canio Buonavoglia, Nicola Decaro

**Affiliations:** ^1^Department of Veterinary Medicine, University of Milan, Lodi, Italy; ^2^Ambulatorio Veterinario Beccaglia, Lissone, Italy; ^3^Department of Veterinary Medicine, University of Bari, Bari, Italy

**Keywords:** canine parvovirus, dog, haemagglutination inhibition test, in-clinic ELISA test, maternally-derived antibodies, vaccination

## Abstract

Canine parvovirus (CPV) is one of the most common causes of mortality in puppies worldwide. Protection against CPV infection is based on vaccination, but maternally-derived antibodies (MDA) can interfere with vaccination. The aim of this study was to evaluate the applicability of an in-clinic ELISA test to assess the CPV MDA in unvaccinated puppies and CPV antibodies in bitches, comparing the results with the gold standard haemagglutination inhibition (HI) test. Serum samples of 136 unvaccinated puppies were tested, along with sera of 16 vaccinated bitches. Five unvaccinated puppies were retested after vaccination. Both assays showed that the 16 vaccinated bitches had protective antibody levels against CPV. Conversely, significant discrepancies were observed for the MDA titers in unvaccinated puppies. Protective MDA titers were observed in 91.9% puppies using HI and in 40.4% by the in-clinic ELISA test, and only the latter one showed a decrease of MDA titers and percentages of protected puppies after the first weeks of age. Vaccination of five puppies with high HI and low in-clinic ELISA MDA titers resulted in seroconversion. Our results confirm the reliability of the in-clinic ELISA test in determining protective antibodies against CPV in adult dogs. Our findings also suggest that the in-clinic ELISA test kit may also be a useful tool to detect and quantify CPV MDA, thus allowing prediction of the best time to vaccinate puppies and reduction of the rate of vaccination failures due to interference by maternally-derived antibodies.

## Introduction

Canine parvovirus (CPV) is one of the most common causes of mortality in puppies (1). The virus is highly contagious and relatively stable in the environment, causing high morbidity in dogs worldwide. Dogs can be infected at any age, but puppies between 6 weeks and 6 months of age are more commonly infected, showing a more severe disease (1). In puppies, maternally-derived antibody (MDA) titers ≥1:80 are considered protective against CPV infection in the first weeks of life (2–4). After the first weeks of age, vaccination is the main method to control the disease worldwide (5).

The World Small Animal Veterinary Association (WSAVA) “Guidelines for vaccination of dogs and cats” recommend that all dogs should be vaccinated, whenever possible, not only to prevent individual infections but also to assure herd immunity and to reduce the prevalence of the disease (6). However, several factors can interfere with an adequate immune response and result in vaccination failure. In puppies, MDA are one of the major factors that can interfere with an immune response to vaccination. According to previous studies, MDA titers ≥1:20 are reported to cause a vaccination failure against CPV (2, 7–9).

CPV MDA vanish with a linear decrease during the post-birth period and their half-life is about 9–10 days (2, 10, 11). In most puppies, MDA decline by 8–12 weeks of age to a level that allows vaccination. Absence of MDA is reported by 10–14 weeks of age (2, 12).

It is not possible to accurately predict the first vaccination time because different MDA titers and kinetics have been reported in puppies, depending on vaccination status of bitches, magnitude of colostrum intake and environmental infective pressure (11, 13). To overcome MDA vaccination interference, administration of initial core vaccination in puppies at 6–8 weeks of age, then every 3–4 weeks until 16 weeks of age or older is recommended by WSAVA guidelines (6). Optimization of vaccination protocols in puppies is recommended and should rely on each puppy's individual needs (11, 14).

It would be important to know MDA titers in puppies in order to reduce interference with vaccination and consequently vaccination failures or, on the other hand, avoid unnecessary vaccinations. Serological testing has been introduced in veterinary practices to determine CPV seroprotection in dogs to assess revaccination requirements (3, 15). The gold standard test for detection and titration of CPV post-infection and/or post-vaccination antibodies in adult dogs is the haemagglutination inhibition (HI) test that has to be performed in specialist diagnostic laboratories (16). Recently, the WSAVA guidelines also support the use of simple in-practice tests for determination of seroprotection in dogs (6). These kits are quick and easy to use in clinics for the determination of immunity duration in vaccinated and/or infected dogs (17–19) but are not licensed to quantify MDA in unvaccinated puppies.

Given the usefulness of testing CPV MDA titers in unvaccinated puppies and the availability of in-practice test kits, the aim of this study was to evaluate the applicability of an in-clinic ELISA test to determine CPV MDA titers in unvaccinated puppies during their first weeks of life and CPV antibody titers in bitches, comparing the results with the gold standard HI test.

## Materials and Methods

### Animals and Sample Collection

Unvaccinated puppies and vaccinated bitches were included after owner's consent to participate in the study, which was approved by Ethics Committee of the Department of Veterinary Medicine, University of Bari (approval number 10/17).

One-hundred and thirty-six puppies and 16 bitches (8 were mothers of the tested puppies) were analyzed in this study, for a total of 152 dogs. Puppies were from 40 litters, ranging from 1 to 11 puppies per litter. Sixty puppies were females and 76 were males. Sixteen animals were <40 days of age, 76 were between 40 and 50 days of age and 44 were >50 days of age. The median age was 47 days. Puppies were from 21 different breeds. Puppies were from small (*n* = 19), medium (*n* = 51), and large (*n* = 66) breed sizes. All the 136 puppies had never been vaccinated. Moreover, 5 unvaccinated puppies were retested after being vaccinated with a trivalent MLV vaccine (Nobivac CEP, MSD) (against CPV, CDV, and CAdV-1 infections). Vaccine was administered to the puppies on the first day of sample collection.

Bitches (*n* = 16) were between 1 and 8 years of age. The median age was 3 years. They were from 12 different breeds and from small (*n* = 3), medium (*n* = 5), and large (*n* = 8) breed sizes. Fourteen bitches were tested between 40 and 50 days of gestation while the other two were tested during the post-partum period with their puppies. All 15 bitches were repeatedly vaccinated starting from 5 months (end of the initial puppy vaccination) till 4 years of age, generally once a year. One cross-breed bitch from a kennel had never been vaccinated and was infected by CPV one week post-partum. Her puppies (*n* = 6), were promptly taken away and remained healthy. Their sera (mother and puppies) were collected 45 days post-partum.

When possible, puppies were retested after the first vaccination. Animals were sampled during 2017 by veterinarians in different Italian clinics, breeding kennels, and animal shelters. Data pertaining to vaccination history and other relevant clinical details were recorded for each dog. Puppies were classified in three age categories: <40 days of age, 40–50 days of age, and >50 days of age. Breeds were classified in small (<10 kg), medium (10–25 kg), and large (>25 kg) size.

Blood samples (1 mL) were collected by cephalic venepuncture from each animal. Samples were immediately centrifuged (1,000 × g for 10 min) and sera were separated and stored at −20°C until analysis.

### In-clinic ELISA Test

Each serum sample was tested using an in-clinic ELISA test (Canine VacciCheck Antibody Test Kit, Biogal, supplied in Italy by Agrolabo), following the manufacturer's instruction. The kit is a rapid dot-ELISA-based system licensed to determine the titer of antibodies against canine adenovirus type 1 (CAdV-1), canine parvovirus (CPV), and canine distemper virus (CDV) antigens. The test kit has been approved by some official agencies and has been used in UK, Israel, and India to evaluate CPV antibodies in dogs (18–21).

The concentration of CPV antibodies in serum samples was defined by the color intensity of the spots measured in “S” units, on a scale from 0 to 6. An S value of 3 (S3) was standardized by the manufacturer to be the equivalence of a 1:80 CPV serum antibody titer by the HI test. As per the information provided by the manufacturer, S units from 0 to 6 corresponds to <1:20, 1:20, 1:40, 1:80, 1:160, 1:320, and 1:640 titer, respectively. Antibody titers ≥1:80 were indicative of protective levels of antibodies to CPV.

### Haemagglutination Inhibition (HI) Test

Serum samples were also subjected to the HI test. Antibody testing was carried out as previously described, with minor modifications (16). The tests were performed at +4°C in 96-well V-plates, using 6–8 haemagglutination units of CPV-2b antigen (22) and 1% porcine erythrocytes. Serial 2-fold serum dilutions were made in phosphate-buffered saline, starting from a 1:10 dilution. Results were read after about 2–4 h at +4°C. The HI titer was indicated as the highest serum dilution completely inhibiting viral haemagglutination. Antibody titers ≥1:80 were indicative of protective levels of antibodies to CPV. As positive controls sera we used known sera from another work already published (23).

### Data Analysis

Statistical analysis was performed using Graph Pad Prism 6, GraphPad Software (La Jolla, CA, USA), and EpiTools Epidemiological Calculators^1^ (24). To compare the validity of in-clinic ELISA with that of haemagglutination inhibition a Spearman correlation test (for not normally distributed data; Shapiro-Wilk test) was used, considering statistically significant value of *p* < 0.05, and a linear regression analyses was also performed. A Pearson's Chi-square test was used to assess the relationship between the presence of protective MDA titers (obtained by in-clinic ELISA or by HI tests) and independent variables such as gender, age, and breed size. A *p* < 0.05 was considered as statistically significant. The relative sensitivity and specificity of in-clinic ELISA were determined with Epitools epidemiological calculators software (epitools.ausvet.com.au) by comparison with the results of HI test (gold standard).

Results of the gold standard (HI) assays were compared with results of the in-clinic ELISA to determine measures of the diagnostic performance of the assay.

## Results

### CPV Antibody Titers and Comparison Between In-clinic ELISA and HI Tests

Considering a MDA titer ≥1:80 as indicative of protection against CPV infection in both tests, the overall percentage of puppies with protective MDA was 40.4% (55/136) using in-clinic ELISA test and 91.9% (125/136) using HI test. Comparison of protective results (MDA titer ≥1:80) obtained in unvaccinated puppies by in-clinic ELISA and HI testing are reported in Table 1.

**Table 1:** Comparison of in-clinic ELISA test and HI test in detecting MDA (titer ≥1:80) in unvaccinated puppies.

	**Positive HI test**	**Negative HI test**	**Total**
**Positive** in-clinic ELISA test	52	3	55 (40.4%)
**Negative** in-clinic ELISA test	73	8	81 (59.6%)
**Total puppies**	125 (91.9%)	11 (8.1%)	136

**Figure 1:**
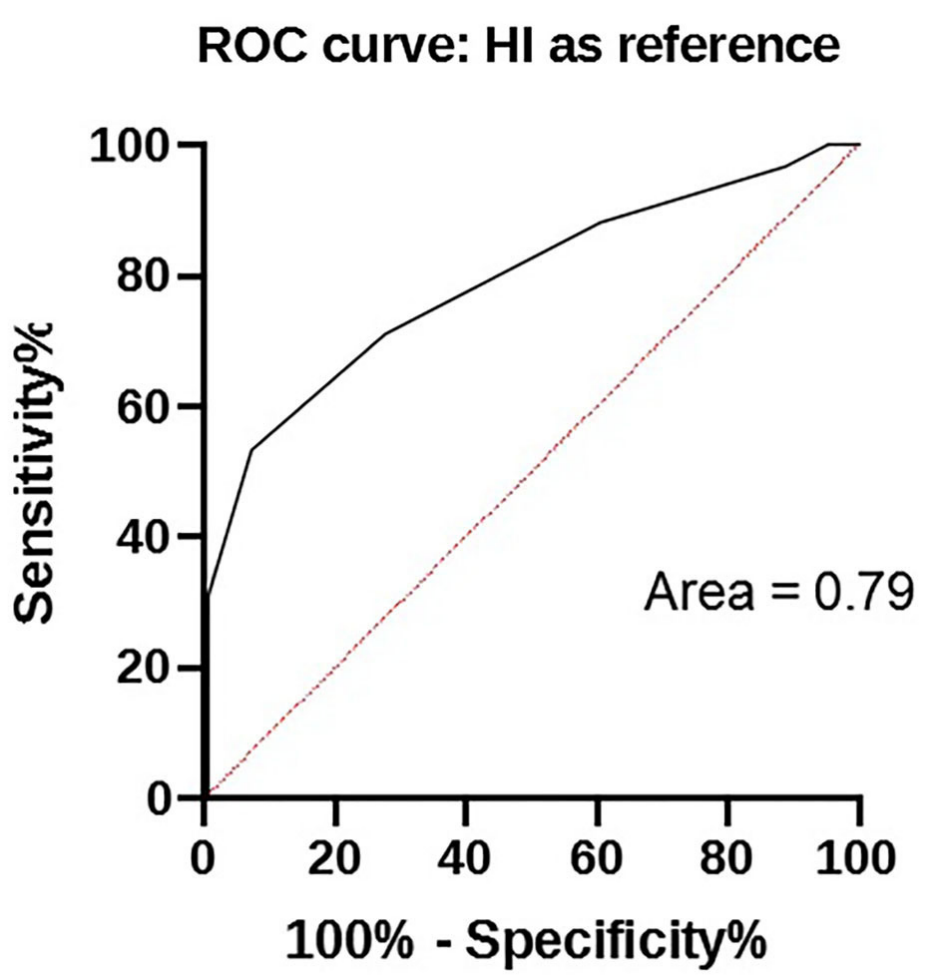
ROC curve between in-clinic ELISA test and HI titers of samples.

The results of HI and in-clinic ELISA are given in Table 2 and relationship exist between these two tests analyzed by ROC is depicted in Figure 1.

**Table 2:** Relative sensitivity and specificity of in-clinic ELISA in comparison to HI test.

		**HI**	
		**Positive**	**Negative**
**VacciCheck**	**Positive**	68	3
	**Negative**	69	12

*Sensitivity = 49.64%*.

*Specificity = 80%*.

**Figure 2:**
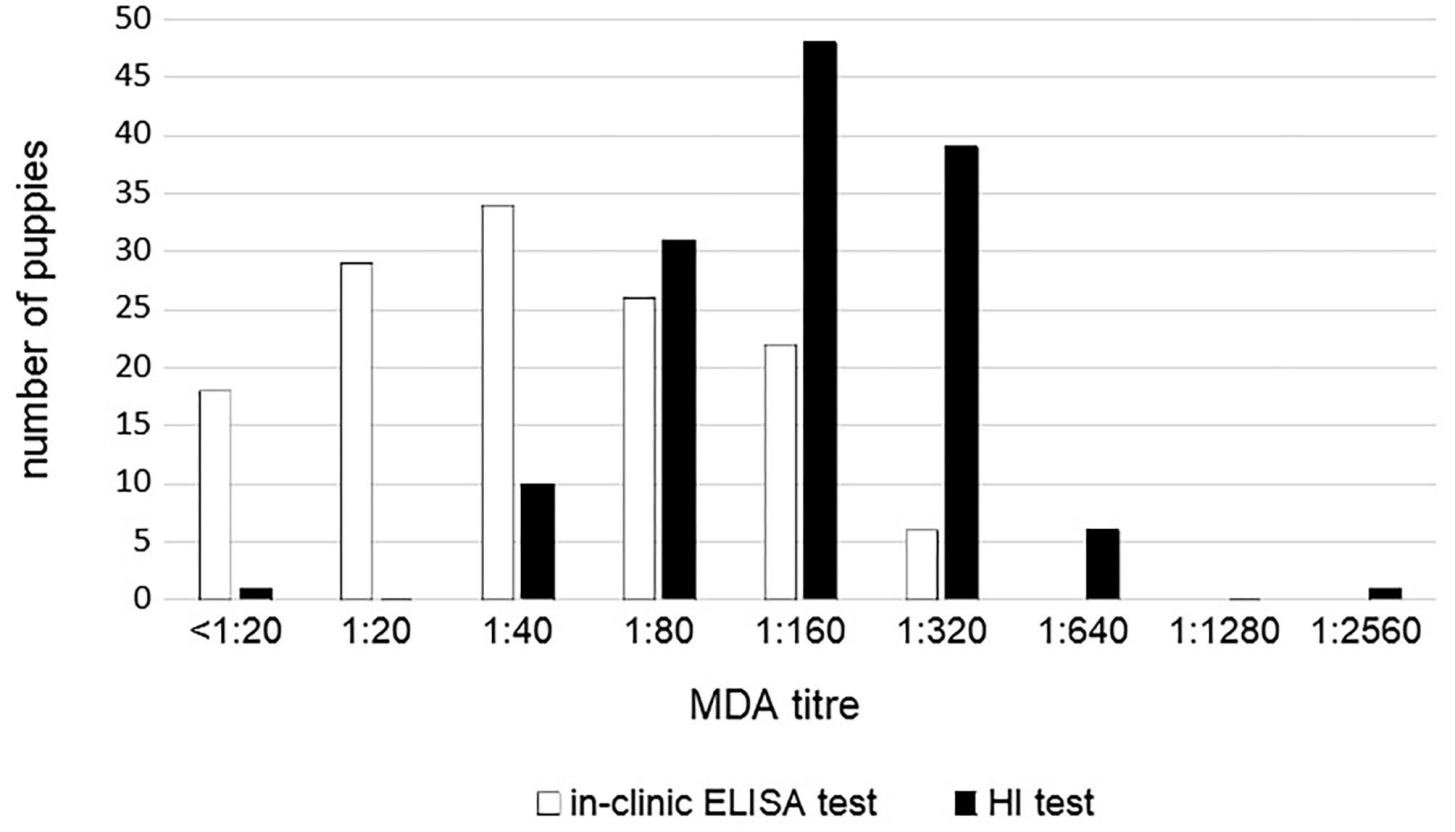
CPV MDA titers in 136 unvaccinated puppies according to the in-clinic ELISA and HI tests results.

MDA titers displayed large variability among puppies and between the two tests. MDA titers ranged from <1:20 to 1:320 using in-clinic ELISA test and from 1:10 to 1:2,560 using HI test (Figure 2). However, the two tests appear to be strictly correlated (*p* < 0.0001).

The results of protected puppies (MDA titer ≥1:80) and antibody titers according to gender, age, and breed size are reported in Table 3.

**Table 3:** Puppies with protective CPV MDA titer (≥1:80) and mean CPV MDA titer detected using in-clinic ELISA test and HI test according to gender, age, and breed size.

		**No. puppies with protective MDA titer (%)**	
	**No. puppies**	**In-clinic ELISA test**	**HI test**
**Gender**			
Female	60	24 (40)	51 (85)^a^
Male	76	31 (40)	74 (97)^a^
**Age**			
<40 days old	16	4 (25)	16 (100)
40-50 days old	76	35 (46)	65 (85)^b^
>50 days old	44	16 (36)	44 (100)^b^
**Breed size**			
Small	19	3 (16)	19 (100)
Medium	51	24 (47)	46 (90)^c^
Large	66	28 (42)	66 (100)^c^

a, b, c *Significant difference between categories of the same variable (p < 0.05)*.

Different MDA titers in puppies from the same litter were observed in majority of the litters. In 11 and 9 litters, puppies from the same litter had the same MDA titer as detected by both HI and in-clinic ELISA test. Both tests always reported different MDA titers between bitches and their offspring.

HI test revealed significant differences in the presence of protective MDA between male and female puppies, with male puppies being significantly more protected than female puppies (*p* = 0.021). Presence of HI protective MDA were significantly lower in 40–50 days old puppies compared with older (>50 days old) puppies (*p* = 0.02). Presence of HI protective MDA were significantly lower in puppies from medium size breeds compared with large size breed (*p* = 0.032). No other significant differences were observed for HI test results. No significant differences were observed between the result of the in-clinic ELISA test and the variables analyzed (gender, age, and breed size) (Table 3).

The MDA and active antibody titers obtained by both tests in 5 puppies tested before and after their first vaccination are shown in Table 4. Before the first vaccination, no puppy had protective titers (all of them presented titers ≤ 1:20) by the in-clinic ELISA test, and this result was in contrast with HI test, which estimated a percentage of 100% of protected puppies. After the first vaccination all puppies seroconverted and became protected as assessed by both tests.

**Table 4:** CPV MDA and active antibody titers detected using in-clinic ELISA test and HI test before and after administration of the first vaccination in 5 puppies.

	**Before vaccination**		**Post-vaccination**	
	**MDA titer**		**Antibody titer**	
**Puppy ID**	**In-clinic-ELISA**	**HI**	**In-clinic ELISA**	**HI**
1	<1:20	1:160	>1:640	1:2,560
2	1:20	1:320	1:80	1:2,560
3	1:20	1:160	1:160	1:1,280
4	1:20	1:640	1:320	1:1,280
5	1:20	1:640	1:80	1:1,280

All the 16 bitches in the study resulted highly protected by both assays. Antibody titers ranged from 1:80 to 1:640 using in-clinic ELISA test and from 1:160 to 1:5,120 using HI test.

The antibody titers of the unvaccinated kennel bitch that was infected with CPV one week post-partum and the MDA titers of her offspring obtained by both tests are shown in Table 5. Forty-five days post-partum, the bitch presented a protective CPV antibody titer due to the previous CPV infection, as detected by both tests; conversely, no puppy was positive using the in-clinic-ELISA test, while 5 of 6 puppies resulted protected by HI.

**Table 5:** Antibody titers against CPV of 6 puppies and their unvaccinated mother.

**Dog ID**	**In-clinic ELISA titer**	**HI titer**
Bitch A	1:160	1:320
Puppy A1	<1:20	1:80
Puppy A2	<1:20	1:80
Puppy A3	<1:20	1:80
Puppy A4	<1:20	1:40
Puppy A5	<1:20	1:80
Puppy A6	1:20	1:80

*The bitch was infected in kennel by CPV one week post-partum and puppies were promptly separated from the dam and remained healthy. Blood samples were collected 45 days post-partum from the puppies and the bitch*.

## Discussion

MDA are known to be a two-edged sword in puppies. MDA are essential for protection against CPV infection but in high concentrations it may cause vaccination failures in puppies. In this study, we evaluated the applicability of an in-clinic ELISA test to assess MDA level in puppies under field conditions in comparison with the gold standard HI test.

Both VacciCheck and HI titers are considered protective if ≥1:80. As stated by Taguchi et al. (25), it is possible to consider a protective titer ≥1:40 using CPV-2b in HI test, as demonstrated in a challenge infection study using a Japanese CPV-2b-based vaccine (Rescamune). In our study, only 10 out of 136 puppies displayed HI MDA titers of 1:40, and according to Taguchi et al. (25) even these puppies could be considered protected against infection by a CPV field strain, thus resulting in a total of 135/136 animals with protective levels of MDA as assessed by HI test (the last one had a titer of 1:10 and then was surely unprotected). However, in the recent review of Chastant and Mila (26) regarding passive immune transfer (PIT) in puppies, adequate PIT was defined as IgG concentration >2.3 g/L for general immunity and CPV-2-antibody titer >1:80 for specific immunity evaluation, independently of CPV-2 strain (26).

The in-clinic ELISA test is commonly used to detect specific CPV post-vaccination/infection antibodies in dogs. In the analyzed bitches, the 100% overall accuracy of the in-clinic ELISA test compared to the HI test to detect protective titers in all the vaccinated adult dogs was expected, since the dogs had been vaccinated within a maximum 4 year-period prior sampling. Vaccination administered within 3-years is considered protective in adult dogs and protective antibody titers have been reported in dogs even after longer periods (19, 27). The results of this study confirm previous findings that indicate the reliability of the in-clinic ELISA test for detection of protective antibodies against CPV in adult dogs (18). Even if antibody titers were not perfectly the same, the higher antibody titers detected using HI test compared to in-clinic ELISA test were previously reported for the HI titers >1:1,280 (17). The in-clinic ELISA test was not able to determine antibody titers in the HI range ≥1:640, indicating that the gold standard test is more reliable in detecting very high antibody titers in dogs after vaccination and/or infection. This limitation is not considered important because titers in the high range indicate protective levels of immunity (17). As reported by Thomas et al. (28), quite low and high HI titers may not have good correlations with any other serological test for the quantification of CPV specific antibodies. This was taken into account while analyzing our results, but in our case no differences were found when those values were eliminated.

Although HI internal control was used, a possible incorrect control titration could have been a bias in the subsequent antibody titration. In fact, as suggested by Senda et al. (29) also small changes in the technique could strongly affect results. Consequently, our HI test might be overestimating puppy antibody titers and be the main cause of the observed discrepancy. Only using true negative sera, it would be possible to increase HI accuracy.

The high percentage (91.9%) of protection and the high MDA titers (1:2,560) by HI test were not expected in puppies, considering the linear decrease of MDA in the first weeks of age (11, 30). Moreover, results obtained by HI test showed a constantly and unexpected highly significant protection of puppies in older age groups (100% puppies >50 days of age with protective MDA titers). According to the expected decline of MDA in the first weeks after birth (2, 10, 11), only the in-clinic ELISA test showed a decrease of MDA titers and percentages of protected puppies starting from 40 days of age (corresponding to ≥6 weeks old puppies).

Compared to older puppies, higher protective MDA titers and prevalence of protected puppies were expected in younger ones (<40 days of age, corresponding to ≤ 6 weeks old puppies). HI test identified 100% of puppies <40 days old as MDA protected. However, protection of all the puppies after 6 weeks from birth is not likely, as demonstrated by lower prevalence previously reported in 6 weeks old puppies (11). The lower protection in puppies ≤ 6 weeks old (<40 days) compared to older ones, as observed by the in-clinic ELISA test, may be due to puppies' features. Anamnestic data revealed that the 16 puppies <40 days old were Dobermanns, Rottweilers and Bull Terriers. These breeds are suspected to be genetically low-responder breeds, thus failing to develop an antibody response after repeated revaccination (6, 31). It is possible that these puppies did not receive adequate MDA because of the low quality of colostrum produced by the bitches due to the inability to develop an adequate antibody response after vaccination. Unfortunately, comparison with puppies <40 days old of other breeds and/or with older Dobermanns, Rottweilers and Bull Terriers puppies was not possible because they were not sampled in this work and further investigations are needed.

The significantly higher percentage of male puppies with protective HI MDA compared to females was not expected and gender being a factor linked to differences in maternal colostrum ingestion in the first day of life has not been reported. The significantly higher percentage of large size breed puppies with protective HI MDA compared to medium size breed was unexpected. An in direct correlation between the duration of MDA and the growth rate of the animal was previously observed, with slow-growth breeds (small and medium size breeds) eliminating their MDA more slowly than rapid growth-breeds (large and giant size breeds) (7).

Differences in MDA titers in bitches and puppies from the same litter were previously reported (17), probably linked to differences in colostrum intake among puppies (7).

CPV antibody titers of the puppies of the unvaccinated bitch infected by CPV one week after delivery also showed discrepancies between the two tests. Puppies were promptly separated from their mother and remained healthy. As expected, by in-clinic ELISA test all puppies presented MDA titers below the protective titer, whereas the HI test showed that 5 of the 6 puppies had protective MDA titers against CPV. Regarding the dam, as a consequence of the CPV infection, specific antibodies protective titers were detected by both tests at 45 days post-partum. The results of the in-clinic-ELISA test seem to be more reliable because these puppies, promptly taken away from the infected mother, remained healthy and did not shed CPV, so that they could not have neither MDA from their unvaccinated mother nor protective antibody titers due to an active immunization.

Discrepancies in results of the two tests in the 5 puppies tested before and after vaccination were also observed. Before the first vaccination the in-clinic ELISA test showed the absence of protective MDA titers in puppies, whereas HI estimated a 100% of puppies having protective MDA. After the first vaccination, all puppies seroconverted and protective antibodies were observed by both tests. Post-vaccination titers seem to support the reliability of the in-clinic ELISA test: in fact, according to previous studies, only puppies with low MDA titers (<1:20) are supposed to develop an appropriate immunity after the first vaccination (2, 7). However, in some circumstances, CPV seroconversion has been observed even in the presence of higher MDA levels (32). Even though the sample is too small, the final result gives a clear indication of the importance of both tests, and further studies are needed.

Overall, our results are indicative of the reliability of the in-clinic ELISA test to detect MDA in puppies and at the same time account for a lower specificity of HI test in determining MDA levels.

Regardless the serological test used, a practical approach may be suggested to overcome the difficulties and expensiveness related to the theoretical possibility to repeatedly sample and test young puppies in order to monitor the decline of MDA and decide the first vaccination time. Instead of repeated sampling, puppies might be tested once for MDA titers, at an age of 6 weeks. Decline of MDA may be subsequently estimated considering a CPV antibody half-life of 9–10 days and vaccination may therefore be scheduled when MDA estimated titers are <1:20 (33).

## Conclusions

The present study reveals the utility of an in-clinic ELISA test in detecting protective antibodies against CPV in adult dogs in comparison with the gold standard HI test. However, discrepancy could be observed between the tests in determining the CPV MDA antibodies in puppies. Only the in-clinic ELISA test showed a decline in MDA titers in older puppies as compared with HI, thus suggesting that this in-clinic ELISA test can be used as a specific and sensitive tool to determine MDA in unvaccinated puppies. This allows the prediction of the best time of vaccination, thus reducing the rate of vaccination failures.

## Data Availability Statement

The original contributions presented in the study are included in the article/supplementary material, further inquiries can be directed to the corresponding author/s.

## Author Contributions

PDA and ND designed this study. PDA, SL, RC, MB, CD, AC, GA, and CB performed the experimental analysis. JF performed the statistical analysis. JF, RC, PDA, and ND drafted and revised the manuscript. All authors read and approved the final manuscript.

## Conflict of Interest

The authors declare that the research was conducted in the absence of any commercial or financial relationships that could be construed as a potential conflict of interest.

## Footnotes

^1^Freely available at http://epitools.ausvet.com.au
